# Supplementary material for: Type 2 Diabetes Prevention Focused on Normalization of Glycemia: A Two-Year Pilot Study
Source: Nutrients. 2021 Feb 26;13(3):749. doi: 10.3390/nu13030749 (PMC7996820; doi:10.3390/nu13030749)
Supplement: Supplementary file 1 [file nutrients-13-00749-s001.pdf]

Supplemental Material

McKenzie AL et al. Type 2 diabetes prevention focused on regression to normoglycemia: a two-year pilot study.

Supplemental Figure S1. Participant Flow Diagram.

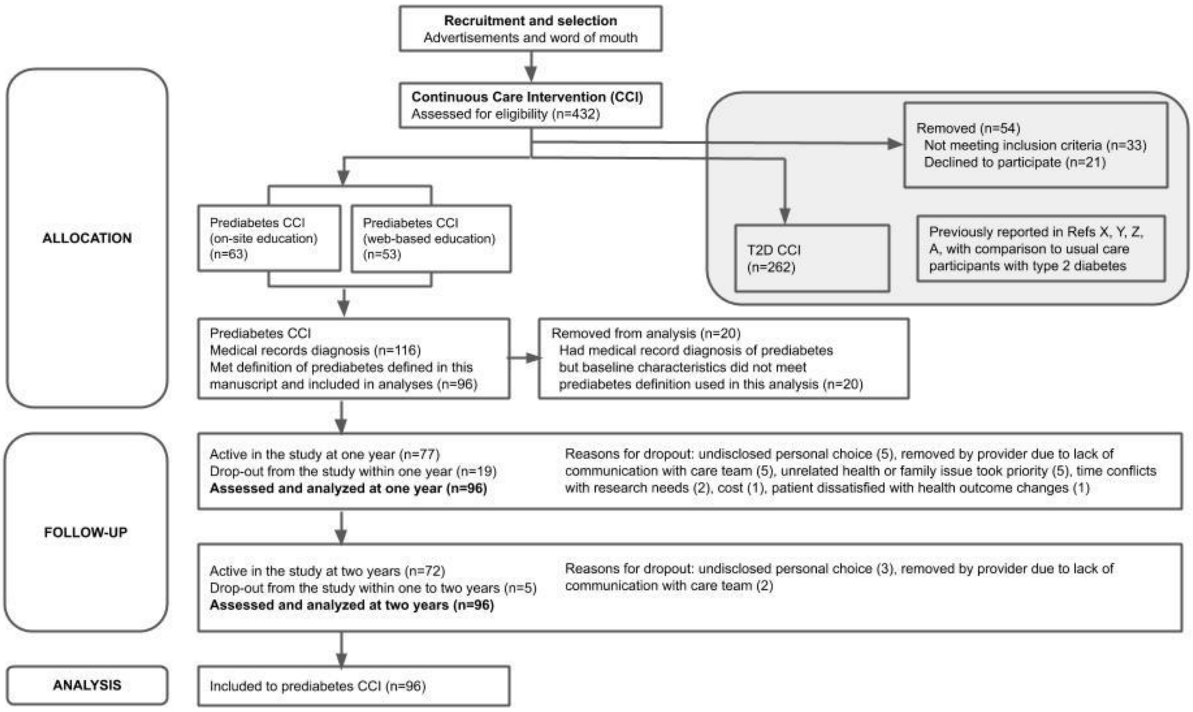

Supplemental Table S1. Baseline characteristics of participants who selected on-site versus web-based education delivery.

	**On-site Education**		**Web-based Education**		
	n	Mean (SD)	n	Mean (SD)	*P*
Age (y)	54	53.0 (10.3)	42	50.1 (8.3)	0.140
Female (%)	54	0.89 (0.32)	42	0.69 (0.47)	0.021
African American (%)	54	0.06 (0.23)	42	0.02 (0.15)	0.445
HbA1c (%)	54	5.93 (0.18)	42	5.96 (0.24)	0.600
HbA1c (mmol/mol)	54	41.3 (2.0)	42	41.6 (2.6)	0.600
Fasting glucose (mmol/L)	53	6.13 (0.84)	42	6.05 (0.85)	0.660
Fasting insulin (pmol/L)	50	171.54 (94.45)	40	202.79 (212.52)	0.393
SBP (mmHg)	54	129.5 (13.9)	41	130.5 (12.9)	0.736
DBP (mmHg)	54	82.1 (7.9)	41	83.4 (7.9)	0.430
Weight (kg)	54	105.87 (23.54)	42	114.9 (25.63)	0.081
BMI (kg/m^2)	54	38.5 (6.4)	42	40.0 (8.2)	0.299
Waist circumference (cm)	41	118.36 (16.51)	33	121.41 (14.73)	0.404
HDL-cholesterol (mmol/L)	50	1.31 (0.37)	40	1.20 (0.32)	0.122
Triglycerides (mmol/L)	50	1.82 (0.83)	40	1.83 (0.92)	0.966
ALT (µkat/L)	53	0.46 (0.28)	42	0.54 (0.39)	0.240
AST (µkat/L)	53	0.40 (0.32)	42	0.42 (0.31)	0.786
NAFLD-Liver Fat Score	50	2.1 (2.4)	39	2.9 (4.9)	0.348

Note: n indicates the available data at the time point. Statistical significance is indicated by $P<0.003$ following Bonferroni correction for multiple comparisons. Abbreviations: SBP, systolic blood pressure; DBP, diastolic blood pressure; HDL, high density lipoprotein; ALT, alanine aminotransferase; AST, aspartate aminotransferase, NAFLD, non-alcoholic fatty liver disease.

Supplemental Table S2. Characteristics of participants who selected on-site versus web-based education delivery after two-years treatment.

	**On-site Education**		**Web-based Education**		
	n	Mean±SE	n	Mean±SE	*P*
HbA1c (%)	37	5.73±0.04	27	5.73±0.04	0.988
HbA1c (mmol/mol)	37	39.3±0.4	27	39.3±0.4	0.988
Fasting glucose (mmol/L)	37	5.73±0.10	26	5.52±0.11	0.129
Fasting insulin (pmol/L)	35	111.12±9.03	23	94.45±10.42	0.192
SBP (mmHg)	27	127.8±2.0	21	126.7±2.2	0.641
DBP (mmHg)	27	80.6±1.3	21	80.4±1.4	0.873
Weight (kg)	42	95.57±2.54	30	99.25±2.90	0.349
BMI (kg/m^2)	42	34.1±0.8	30	35.3±1.0	0.340
Waist circumference (cm)	21	111.25±3.05	21	110.74±3.30	0.875
HDL-cholesterol (mmol/L)	35	1.47±0.06	23	1.45±0.06	0.780
Triglycerides (mmol/L)	35	1.28±0.10	23	1.29±0.12	0.953
ALT (µkat/L)	37	0.35±0.02	26	0.38±0.03	0.388
AST (µkat/L)	37	0.34±0.01	26	0.33±0.02	0.530
NAFLD-Liver Fat Score	25	-0.33±0.30	17	-0.39±0.32	0.871

Note: n indicates the available data at the time point. Statistical significance is indicated by $P<0.003$ following Bonferroni correction for multiple comparisons. Abbreviations: SBP, systolic blood pressure; DBP, diastolic blood pressure; HDL, high density lipoprotein; ALT, alanine aminotransferase; AST, aspartate aminotransferase, NAFLD, non-alcoholic fatty liver disease.

Supplemental Table S3. Baseline characteristics of participants and comparison of completers and dropouts.

	All		Completers with data		Dropouts or missing data		
	n	Mean (SD)	n	Mean (SD)	n	Mean (SD)	*P*
Age (years)	96	51.75(9.52)	72	52.50(8.66)	24	49.50(11.68)	0.256
Female (%)	96	80.0(40.1)	72	82.0(38.7)	24	75.0(44.0)	0.465
African American (%)	96	4.0(20.1)	72	6.0(23.2)	24	0.0(0.0)	0.045
HbA1c (%)	96	5.94 (0.21)	72	5.93(0.20)	24	5.98(0.23)	0.365
HbA1c (mmol/mol)	96	41.4 (2.3)	72	41.3 (2.2)	24	41.8 (2.5)	0.365
Fasting glucose (mmol/L)	95	6.09 (0.85)	71	6.15 (0.86)	24	5.92 (0.78)	0.249
Fasting insulin (pmol/L)	90	185.6 (157.8)	67	174.3 (159.5)	23	218.4 (151.2)	0.250
SBP (mmHg)	95	129.9(13.4)	71	130.3(13.2)	24	128.9(14.3)	0.672
DBP (mmHg)	95	82.6(7.9)	71	81.9(7.4)	24	84.7(8.9)	0.143
Weight (kg)	96	109.83 (24.77)	72	108.26 (23.49)	24	115.44 (28.27)	0.284
BMI (kg/m^2)	96	39.14(7.06)	72	38.79(7.48)	24	40.18(6.49)	0.419
Waist circumference (cm)	74	119.66 (15.72)	57	117.93 (14.81)	17	125.48 (17.70)	0.083
HDL-cholesterol (mmol/L)	90	1.26 (0.35)	68	1.29 (0.35)	22	1.16 (0.34)	0.113
Triglycerides (mmol/L)	90	1.83 (0.87)	68	1.74 (0.88)	22	2.11 (0.78)	0.085
ALT (µkat/L)	95	0.50 (0.34)	71	0.48 (0.35)	24	0.55 (0.31)	0.357
AST (µkat/L)	95	0.41 (0.31)	71	0.39 (0.28)	24	0.46 (0.39)	0.365
NAFLD-Liver fat score	89	2.42(3.72)	67	2.09(3.64)	22	3.45(3.84)	0.137

Note: n indicates the available data at the time point. Statistical significance is indicated by $P<0.003$ following Bonferroni correction for multiple comparisons. Abbreviations: SBP, systolic blood pressure; DBP, diastolic blood pressure; HDL, high density lipoprotein; ALT, alanine aminotransferase; AST, aspartate aminotransferase, NAFLD, non-alcoholic fatty liver disease.
